# Supplementary material for: Spiky figures correspond to Japanese names written in square characters
Source: Psychon Bull Rev. 2026 Aug 10;33(6):208. doi: 10.3758/s13423-026-02969-6 (PMC13457275; doi:10.3758/s13423-026-02969-6)
Supplement: Supplementary file 1 [file 13423_2026_2969_MOESM1_ESM.docx]

**Supplemental Materials**

**Data and Script Variables**

For transparency and to facilitate the reproducibility of our analyses using the OSF dataset and R script, please note the correspondence between the variable labels in the dataset and the terminology used in the manuscript. In the dataset, the outline variable is coded as “curve” and “straight,” which correspond to “rounded shapes” and “spiky shapes” in the manuscript, respectively. Similarly, the character variable is coded as “cursive” and “square,” representing “hiragana” and “katakana,” respectively.

**Analyses**

First, we calculated the means and standard deviations for four conditions of character type (cursive: hiragana and square: katakana) and shape outline (straight and curved lines) using the “Rmisc” package (Hope, 2022) on R (R Core Team, 2024).

Next, a linear mixed-effects analysis was conducted to examine how character and shape outline affect the appropriateness ratings of a word as the name for a given shape. Character type and figure outline were used as fixed effects, and participants and words, as random effects. The dependent variable was appropriateness ratings (0-100). Then, we compared models using Akaike Information Criteria (AIC). Ultimately, the following model was used for the analysis: model = lmer (appropriate ~ outline*character + (1 + outline | id) + (1 | word)). We calculated conditional and marginal *R^2^* and standardized coefficients for the final model, and Cohen’s *d* for the post-hoc tests. Degrees of freedom for the fixed-effect estimates were approximated using the Satterthwaite method, whereas degrees of freedom for the post-hoc pairwise comparisons were approximated using the Kenward-Roger method. We used “lme4” (Bates et al., 2015) and “lmerTest” (Kuznetsova et al., 2017) for linear mixed-effects modeling, “emmeans” for post-hoc tests (Lenth, 2025), “parameters” for standardized estimates (Lüdecke et al., 2020), “performance” for R squared (Lüdecke et al., 2021), “dplyr” for data manipulation (Wickham et al., 2023), and “writexl” for exporting results (Ooms, 2024).

**Supplemental Figure 1**

*Examples of Rounded Figures*

*Note.* From left to right, they have 6, 10, and 14 dots, respectively.

**Supplemental Figure 2**

*Examples of Spiky Figures*

*Note.* From left to right, the figures have 6, 10, and 14 dots, respectively.

**References**

Bates, D., Mächler, M., Bolker, B., & Walker, S. (2015). Fitting linear mixed-effects models using lme4. *Journal of Statistical Software, 67*(1), 1–48. https://doi.org/10.18637/jss.v067.i01

Hope, R. M. (2022). *Rmisc: Ryan miscellaneous*. R package version 1.5.1. https://CRAN.R-project.org/package=Rmisc

Kuznetsova, A., Brockhoff, P. B., & Christensen, R. H. B. (2017). lmerTest package: Tests in linear mixed effects models. *Journal of Statistical Software, 82*(13), 1–26. https://doi.org/10.18637/jss.v082.i13

Lenth, R. (2025). *emmeans: Estimated marginal means, aka least-squares means*. R package version 1.10.7. https://CRAN.R-project.org/package=emmeans

Lüdecke, D., Ben-Shachar, M., Patil, I., & Makowski, D. (2020). Extracting, computing and exploring the parameters of statistical models using R. *Journal of Open Source Software, 5*(53), 2445. https://doi.org/10.21105/joss.02445

Lüdecke, D., Ben-Shachar, M. S., Patil, I., Waggoner, P., & Makowski, D. (2021). performance: An R package for assessment, comparison and testing of statistical models. *Journal of Open Source Software, 6*(60), 3139, https://doi.org/10.21105/joss.03139

Ooms, J. (2024). *writexl: Export data frames to excel ‘xlsx’ format*. R package version 1.5.1. https://CRAN.R-project.org/package=writexl.

R Core Team. (2024). *R: A language and environment for statistical computing*. R Foundation for Statistical Computing, Vienna, Austria. https://www.R-project.org/

Wickham, H., François, R., Henry, L., Müller, K., & Vaughan, D. (2023). *dplyr: A grammar of data manipulation*. R package version 1.1.4, https://CRAN.R-project.org/package=dplyr
